# Neurological Emergencies in Refugees

**DOI:** 10.3389/fneur.2018.01088

**Published:** 2018-12-11

**Authors:** Marie P. Brinckmann, Betteke M. van Noort, Christoph Leithner, Christoph J. Ploner

**Affiliations:** ^1^Department of Neurology, Charité-Universitätsmedizin Berlin, Berlin, Germany; ^2^Department of Child and Adolescent Psychiatry, Psychosomatic Medicine and Psychotherapy, Charité-Universitätsmedizin Berlin, Berlin, Germany

**Keywords:** emergency room, emergencies, refugees, migration, forced displacement, multicultural health care

## Abstract

**Objective:** Health care personnel in Europe is increasingly involved in care of displaced persons from non-European countries; we investigated the spectrum of neurological disorders and medical management in refugees presenting to the emergency room (ER) of a German university hospital.

**Methods:** We retrospectively studied ER-patients with refugee status (R-patients) during the peak of the European refugee crisis between July 2015 and February 2016 (*N* = 100). Complaints on admission, medical management and diagnoses at discharge were compared to matched groups of German residents with migrational background (M-patients; *N* = 96) and to native Germans (N-patients; *N* = 95).

**Results:** R-patients were mostly male young adults (75% male; mean age 33.2 years). Headache was the most frequent complaint in all groups (R-patients 38%; M-patients 43%; N-patients 24%). R-patients, however, presented much more often with possible or definite seizures (R-patients 27%; M-patients 9%; N-patients 15%). Initial triage, length of medical history and examination records, utilization of laboratory tests and cranial imaging did not differ between groups. However, time to diagnosis was considerably longer in R-patients (220 min; M-patients 151 min, N-patients 123 min). While strokes and other life-threatening emergencies were rare final diagnoses in R-patients, a substantial proportion was discharged with a diagnosis of non-epileptic seizures or a psychiatric disorder (20%; M-patients 6%; N-patients 7%).

**Conclusions:** Refugee patients present with a spectrum of neurological disorders that not solely results from cultural differences but rather reflects the consequences of forced displacement. ER management of refugees requires more time, language skills and critically depends on psychosomatic/psychiatric expertise.

## Introduction

The growing number of refugees worldwide has developed into a major societal and health-care challenge, even in countries of the European Union (EU). Currently, there are 65.6 million forcibly displaced people worldwide. Among them, 5.6 million refugees are of Syrian and Afghan origin (1). In the recent few years, emergency rooms (ER) in the EU have been facing rising numbers of patients from Syria, Afghanistan, and Iraq seeking asylum—in particular in Germany (2). For example, in 2015, an estimated 90,000 refugees arrived in Berlin. Although refugee numbers from war-ridden countries have been slowly decreasing since the beginning of 2016, migration under precarious circumstances continues (3, 4). Health care personnel delivering care for refugees is facing challenges that result both from cultural differences and a lack of familiarity with the physical and psychological consequences of war, of a traumatizing journey and of involuntary relocation (5–7).

Apart from health care problems that are clearly attributable to war trauma, malnutrition, hygiene problems etc., previous studies suggested the possibility that forced migration may moreover induce a distinct spectrum of disorders that is not a mere consequence of cultural differences, but rather a signature of migration itself (8, 9). Several studies have observed that adversity suffered by refugees is associated to a particular range of neuropsychiatric conditions such as post-traumatic stress disorder, depression, and anxiety disorders (8–14). On the other hand, it has been claimed that a different cultural background may already account for significant differences in the spectrum of disorders found in migrants and residents in ERs (15). Since 2015, many hospitals in the EU have released volunteering doctors from regular clinical duties to provide basic medical care in refugee camps. However, data on the medical conditions refugees present with are scarce. It is currently unclear, whether the spectrum of diseases seen by emergency physicians in the EU reflects direct consequences of migration or merely results from cultural and demographical factors.

Here, we took the unique opportunity to gather data from neurological ER-Patients with refugee status in the largest university hospital in Europe. We investigated possible differences between the spectrum of neurological disorders in refugees and in age- and gender-matched controls with immigrant background and in patients with no immigrant background. We focused on the period from July 2015 to February 2016 that corresponded to the peak of refugee arrivals in Germany (16). Our main goal was to investigate whether there is a spectrum of neurological disorders that is directly related to refugee status. In addition, we aimed to characterize the resources necessary for an appropriate neurological emergency care in refugees.

## Methods

### Subjects

For this study, we evaluated all patients with a refugee status who presented with neurological complaints to the ER of the Charité-Universitätsmedizin Berlin between the 1st of July, 2015 and 28th of February, 2016. This period corresponds to the peak of immigration to Germany during the Syrian Civil War. Refugee patients (R) were not selected by region of origin, but rather identified by manually selecting Arab, Pashto, and Dari surnames and screening the corresponding ER records. These three languages are spoken in Syria, Iraq, and Afghanistan, respectively (17), i.e., the homelands of the majority of non-European refugees in Germany in 2015 (18). When a refugee status was not explicitly stated in the documents, the patient was not included. From the insurance status, place of residence and the medical histories it was obvious for each of the included R-group patients, that the duration of stay in Germany did not exceed some weeks prior to admission. Two groups of age- and sex-matched controls were selected from the same time period: A first group that consisted of patients with an immigrant background as identified by an Arab, Pashto or Dari surname and German residency of at least 2 years (M). Patients who did not speak German or with unclear duration of residency in Germany were not included. A second group of controls consisted of patients without immigrant background (N), selected by their German first names and surnames. The study was conducted in accordance with the World Medical Association's Declaration of Helsinki and was approved by the Ethics committee of the Charité—Universitätsmedizin Berlin.

### Demographical and Clinical Variables

The following variables were extracted from digital ER records: age, gender, main symptom on admission to the ER, initial medical triage, presence of an interpreter, compliance, length of medical history, and of physical examination, presence/absence of blood analyses, cranial imaging, and lumbar puncture. Furthermore, we determined diagnosis on discharge from the ER, time to diagnosis, treatment (medication), and the number of patients receiving further in-hospital treatment.

#### Initial Medical Triage

Prioritization for treatment was performed by using the Manchester Triage System (19) in almost all patients (missing values: R-patients 3; M-patients 3; N-patients 4). In the MTS version implemented in our hospital, patients are assigned by nursing staff to one of five possible priority classes on arrival at the ER, ranging from “red” (requires immediate treatment) to “blue” (should see a physician within 120 min). We reasoned that between-group differences of this variable may reveal any positive or negative bias of the nursing staff toward the investigated patient groups.

#### Compliance

Compliance was rated as present or absent. Full compliance was assumed when the patient agreed on all diagnostic procedures and therapeutic suggestions of the ER neurologist. Refusal of diagnostic procedures (e.g., of a lumbar puncture) or of hospitalization was rated as non-compliance.

#### Length of Medical History and Physical Examination

The word counting tool in Microsoft Word was used to determine the length of medical histories and physical examination reports. We reasoned that this variable reflects the diligence of history taking in a given patient and may thus, indirectly, reveal any positive or negative bias of the ER neurologists toward the investigated patent groups.

#### Blood Analyses, CT, MRI, Lumbar Puncture, and Administration of Medication

These variables were rated as absent or present.

#### Time to Diagnosis

We determined the time in minutes from ER arrival, i.e., first log-in of the MTS nurse for patient triage, to the moment of completion of the neurologist's report in the hospital's digital information system.

## Data Analysis

IBM SPSS Statistics 22 was used for data analysis. To evaluate demographic and clinical differences between groups, chi-square tests for homogeneity with *post-hoc* Bonferroni corrected *z*-tests were used. When the sample size assumption was not met, a Fisher's exact test with, when necessary, Monte Carlo simulation (10,000) was conducted instead. For between-group comparisons of continuous variables, one-way ANOVAs with *post-hoc* tests were conducted. To adjust for multiple comparisons, Tukey's honest significance difference or Games-Howell was used. The sample distribution was balanced with an almost equal number of observations in each group, thus one-way ANOVA was considered robust against any non-normality (20, 21).

## Results

### Demographic Data

**Figure 1:**
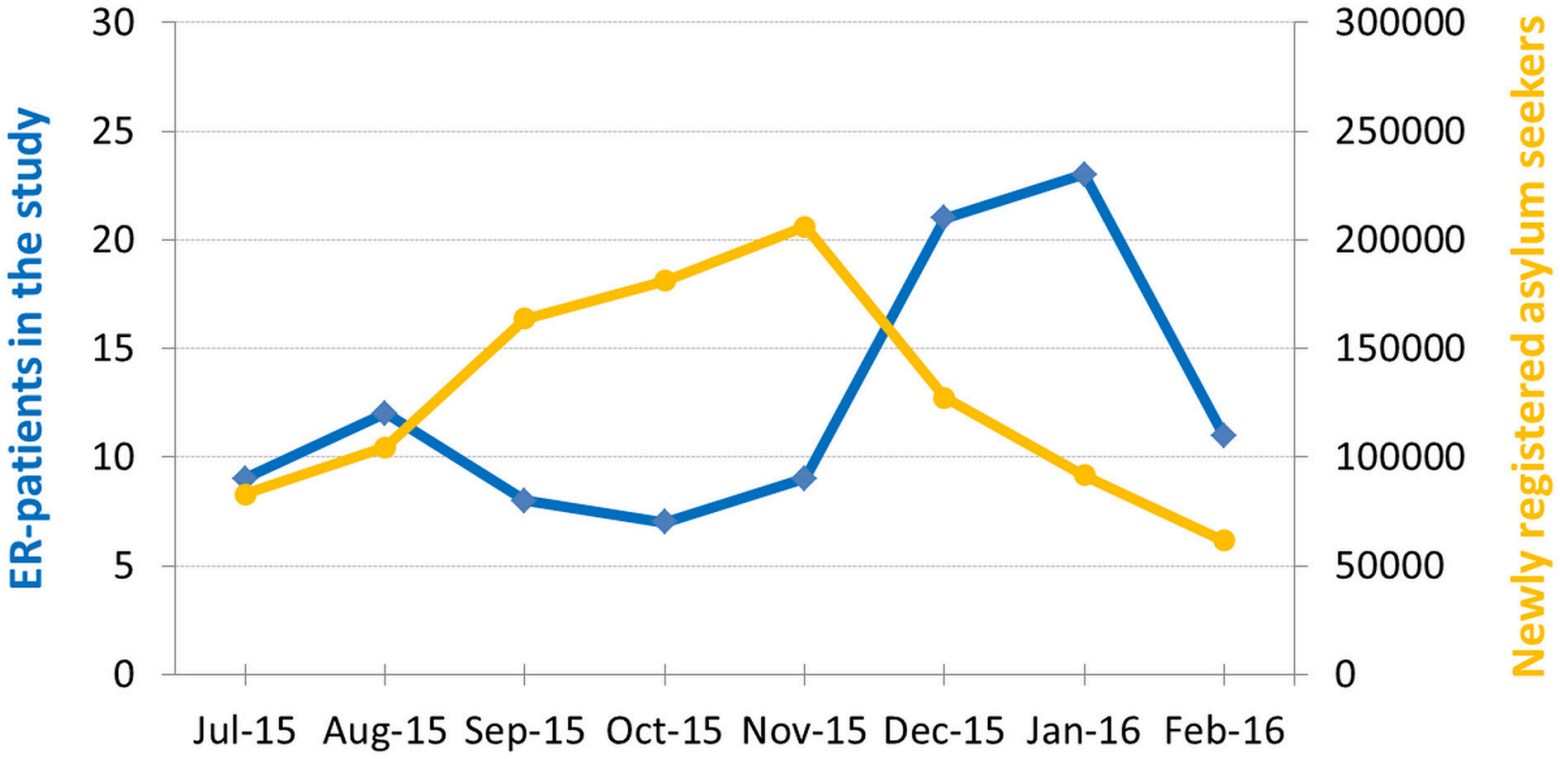
Number of ER-patients with refugee status (blue) and number of first registrations of asylum seekers in Germany (yellow) during the recruitment period (16). Note peak of ER-patients with refugee status immediately after peak of registrations.

We identified 100 refugee patients (R-patients, 75% male, mean age 33.2 years ± 1.3, Table 1) who had presented to the ER with neurological symptoms. ER-Presentation of R-patients was not equally distributed across the study period, but rather showed a peak in January 2016, i.e., shortly after the peak of arrivals of asylum seekers in Germany (Figure 1).

**Table 1:** Demographical data and medical management of patients.

	**Refugee status (R)**	**Migration (M)**	**No migration (N)**	**χ^2^/F**	***df***	***p***	*post-hoc* compa-risons^*^
	***N* = 100**	***N* = 96**	***N* = 95**				
	***n* (%)/M ± SE**	***n* (%)/M ± SE**	***n* (%)/M ± SE**				
Gender (male)	75 (75.0)	60 (62.5)	58 (61.1)	5.2	2	0.075	–
Age	33.2 ± 1.3	34.5 ± 1.2	33.6 ± 1.4	0.3	2	0.777	–
Triage^a^				12.6	-	0.128	–
Immediate: 0 min.	4 (4.1)	0 (0.0)	0 (0.0)				–
Very urgent: 10 min.	15 (15.5)	10 (10.8)	19 (21.3)				–
Urgent: 30 min.	45 (46.4)	51 (54.8)	38 (42.7)				–
Standard: 90 min.	30 (30.9)	30 (32.3)	30 (33.7)				–
Non-urgent: 120 min.	3 (3.1)	2 (2.2)	2 (2.2)				–
Interpreter present^b^	57 (60.0)	11 (11.5)	0 (0.0)	106.5	2	< 0.001	R > M,N M > N
Medical history	102.9 ± 4.8	105.3 ± 5.0	82.2 ± 4.3	7.2	2	0.001	R, M < N
Examination report^c^	84.0 ± 3.6	94.8 ± 4.7	85.0 ± 4.2	2.0	2	0.132	-
Full compliance	89 (89.0)	82 (85.4)	83 (87.4)	0.6	2	0.753	-
Blood analyses	89 (89.0)	91 (94.8)	88 (92.6)	2.3	2	0.315	-
Cranial imaging							
CT	44 (44.0)	31 (32.3)	38 (40.0)	2.9	2	0.234	–
MRI	16 (16.0)	27 (28.1)	29 (30.5)	6.4	2	0.041	R < N
Lumbar puncture	9 (9.0)	15 (15.6)	14 (14.7)	2.2	2	0.326	–
Time to diagnosis (in min.)^d^	220.5 ± 16.1	150.6 ± 13.0	122.6 ± 9.3	14.8	2	< 0.001	R > M, N
Medication in ER	19 (19.0)	31 (32.3)	41 (43.2)	13.3	2	0.001	R < N
Inpatient	28 (28.0)	35 (36.5)	44 (46.8)	7.4	2	0.025	R < N
Repeated ER visit	26 (26.0)	38 (39.6)	31 (32.6)	4.1	2	0.128	-

* *p = 0.05, Bonferroni corrected post-hoc z-tests for chi-square or Tukey's honest significance difference or Games-Howell post-hoc tests for ANOVA*.

a *n_R_ = 97, n_M_ = 93, n_N_ = 89*.

b *n_R_ = 95*.

c *n_N_ = 94*.

d *n_R_ = 99, n_M_ = 94*.

Demographics of R-patients did not correspond to the average distribution of age and gender of neurological ER-patients. Compared to demographical data from a previous study in the same ER (21), R-patients were younger and more often male. We therefore matched M- and N- patients presenting during the same study period as closely as possible for age and gender to R-patients (*p* = 0.777 and *p* = 0.075 difference between groups, Table 1). For the M-group, we identified 96 patients with an immigrant background (63% male, mean age 34.5 years ± 1.2). For the N-group, we found 95 patients without immigrant background fulfilling inclusion criteria for our study (61% male, mean age 33.6 years ± 1.4).

### Triage on Admission

**Figure 2:**
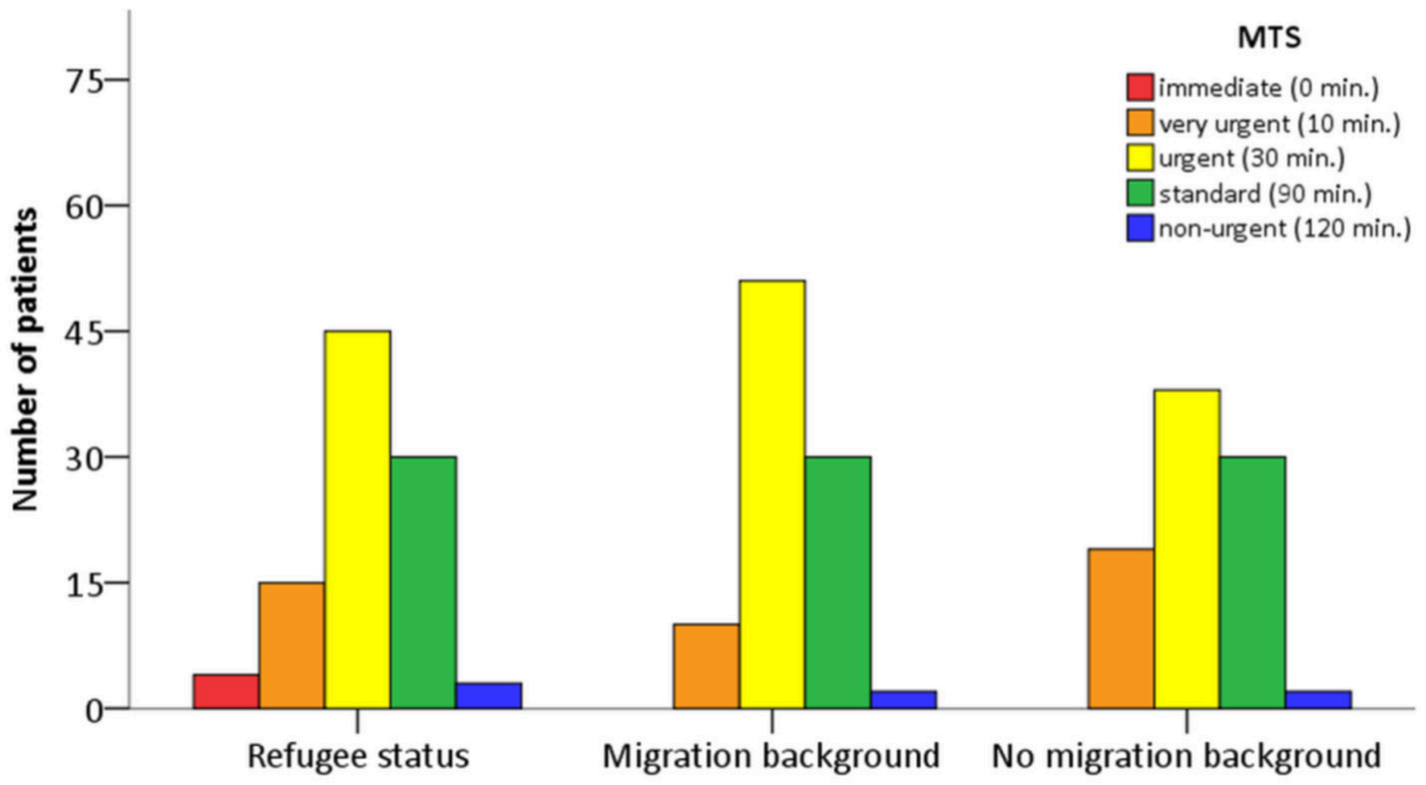
Manchester Triage (MTS) of patient groups on admission.

We found no significant differences in the distribution of MTS classes between groups (Table 1, Figure 2). The majority of patients in either group were assigned to class “yellow” (should see a physician within 30 min) and “green” (should see a physician within 90 min). Thus, on admission, R-patients received a medical prioritization by nursing staff that did not differ from the other two groups.

### Complaints on Admission

Headache was the most frequent complaint in all three patient groups. However, consistent with previous studies of socio-cultural influences on neurological chief complaints (22), this symptom was more frequent in R-patients (37.8%) and M-patients (42.7%) compared to N-patients (24%). The second most common complaint in R-patients was a seizure-like event (25.2%). The frequency of this complaint in R-patients differed significantly from the other groups (M-patients 4.2%, *p* < 0.05; N-patients 7.4%, *p* < 0.05). Dizziness was common and occurred at a similar frequency in all groups (M-patients 18.2%; N-patients 18.9%; R-patients 13.3%). Less frequent complaints on admission were somatosensory impairments, paresis, definite seizures (patients with an established diagnosis of epilepsy), decreased vigilance, cranial nerve disorders (Bell's palsy), and psychiatric disorders (Table 2).

**Table 2:** Neurological complaints on admission and diagnoses at discharge.

	**Refugee status (R)**	**Migration (M)**	**No migration (N)**	**χ^2^**	***p***	*post-hoc* compa-risons^*^
	***N* = 100**	***N* = 96**	***N* = 95**			
	***n* (%)**	***n* (%)**	***n* (%)**			
**Complaints on admission**				45.1^a^	0.001	
Headache	37 (37.8)	41 (42.7)	23 (24.2)			M > N
Dizziness	13 (13.3)	18 (18.2)	18 (18.9)			n.s.
Possible seizure	25 (25.2)	4 (4.2)	7 (7.4)			R > M,N
Definite seizure	2 (2.0)	5 (5.2)	8 (8.4)			n.s.
Paresis	6 (6.1)	4 (4.2)	8 (8.4)			n.s.
Somatosensory impairments	7 (7.1)	9 (9.4)	15 (15.8)			n.s.
Decreased vigilance	5 (5.1)	4 (4.2)	3 (3.2)			n.s.
Cranial nerve disorders	3 (3.1)	10 (10.4)	10 (10.5)			n.s.
Psychiatric disorder	0 (0.0)	0 (0.0)	3 (3.2)			n.s.
Dementia	0 (0.0)	1 (1.0)	0 (0.0)			n.s.
**Diagnoses at discharge**				67.6^a^	0.001
Headaches	*n* = 33	*n* = 34	*n* = 26		
Migraine	5 (5.0)	13 (13.5)	11 (11.6)			n.s.
Tension-type headache	3 (3.0)	8 (8.3)	5 (5.3)			n.s.
Trigeminal-autonomic headaches	3 (3.0)	1 (1.0)	1 (1.1)			n.s.
Parainfectious headache	8 (8.0)	2 (2.1)	5 (5.3)			n.s.
Other symptomatic headaches	8 (8.0)	2 (2.1)	3 (3.2)			n.s.
Headache of unknown etiology	11 (11.0)	8 (8.3)	1 (1.1)			R > N
Seizures and related disorders	*n* = 29	*n* = 13	*n* = 20		
Non-epileptic seizure	11 (11.0)	2 (2.1)	2 (2.1)			R > M,N
Seizure	7 (7.0)	5 (5.2)	7 (7.4)			n.s.
Symptomatic epileptic seizure	4 (4.0)	1 (1.0)	8 (8.4)			M < N
Syncope	7 (7.0)	5 (5.2)	3 (3.2)			n.s.
Other	*n* = 33	*n* = 37	*n* = 42			n.s.
Psychiatric diagnosis	9 (9.0)	4 (4.2)	5 (5.3)			n.s.
Multiple sclerosis	5 (5.0)	5 (5.2)	14 (14.7)			n.s.
Stroke	4 (4.0)	7 (7.3)	12 (12.6)			n.s.
Dizziness of unknown etiology	3 (3.0)	7 (7.3)	4 (4.2)			n.s.
Benign paroxysmal positional vertigo	4 (4.0)	1 (1.0)	1 (1.1)			n.s.
Cranial nerve palsy	1 (1.0)	8 (8.3)	4 (4.2)			n.s.
Other discipline	7 (7.0)	5 (5.2)	2 (2.1)			n.s.
Miscellaneous disorders^b^	5 (5.0)	11 (11.5)	7 (7.4)			n.s.

* *p = 0.05, Bonferroni corrected post-hoc z-tests. n.s, not significant*.

a *Fisher's exact test with Monte Carlo simulation (10,000) when necessary*.

b *metabolic diseases, motoneuron disease, myasthenia gravis, neuritis vestibularis, peripheral nerve affection, pseudotumor cerebri, psychoorganic syndrome*.

### Diagnoses at Discharge

The pattern of diagnoses at discharge from the ER differed significantly between groups [$\chi_{\left(2\right)}^{2}$ = 67.6, *p* = 0.001 Table 2]. A frequent diagnosis in the R-patients was headache of unknown etiology (11.0%). This diagnosis was significantly more often assigned to R-patients than to N-patients (1.1%; *p* < 0.05). Likewise, a diagnosis of non-epileptic (i.e., psychogenic) seizures was significantly more often assigned to R-patients than to M-patients and N-patients (11.0 vs. 2.1 and vs. 2.1%, respectively, both *p* < 0.05). By contrast, symptomatic epileptic seizures occurred less often in R-patients (4.0%) and M-patients (1.0%) compared to N-patients (8.4%). Psychiatric diagnoses other than non-epileptic seizures were seen in 9.0% of R-patients, in 4.2% of M-patients and 5.3% of N-patients. Thus, the combined frequency of non-epileptic seizures and other psychiatric disorders was significantly higher in R-patients (20.0%) than in M-patients (6.3%, *p* < 00.05) and N-patients (7.4%, *p* < 0.05). Conversely, the combined frequency of serious neurological disorders requiring immediate in-hospital treatment (stroke and multiple sclerosis) was significantly lower in R-patients (9.0%) than in N-patients (27,3%, *p* < 0.05), but not different from M-patients (12.5%, *p* > 0.05).

### Length of Medical History and Examination

Medical histories were longer in R- and M-patients compared to N-patients (*p* = 0.006 and *p* = 0.002, respectively, Table 1). By contrast, length of physical examination records did not differ between groups, indicating that refugees received a similar medical attention as the other two groups (Table 1).

### CT, MRI, Blood Analyses, and Lumbar Puncture

Cranial imaging (CT, MRI) was performed at a similar frequency in all three groups (R-patients 53.0%; M-patients 49.0%; N-patients 56.8%; Table 1). However, consistent with the different pattern of diagnoses at discharge from the ER, R-patients received significantly less often MRI (16.0%) compared to N-patients (30.5%; *p* = 0.041). No significant difference in the number of MRIs was found compared to M-patients (28.0%; *p* = 0.057). Blood analyses were performed at a similar frequency across groups (R-patients 89.0%; M-patients 94.8%; N-patients 92.6%; Table 1). Likewise, we found no significant differences between frequencies of lumbar punctures across groups (R-patients 9.0%; M-patients 15.6%; N-patients 11%; Table 1).

### Interpreters

In most R-patients, communication and history taking was not sufficiently possible in English. Thus, an interpreter was present during management of 60.0% of R-patients (29% were professional interpreters), whereas only 11.5% of M-patients were interviewed with the help of an interpreter (2% were professional interpreters) (Table 1). In N-patients, interpreters were not called upon.

### Time to Diagnosis

Although the clinical work-up was similar across groups, time to diagnosis showed large and significant differences. R-patients spent significantly more time in the ER with a mean stay of 220 min compared to 151 min in M-patients and 123 min in N-patients (Table 1).

### Pharmacological Treatment in the ER and Further In-hospital Treatment

R-patients received significantly less often pharmacological treatment in the ER than M-patients and N-patients (19.0% compared to 32.3 and 43.2%, respectively; *p* < 0.05 for both comparisons). Consistent with the different pattern of diagnoses at discharge, R-patients also received less often further in-hospital treatment (28.0%) than N-patients (46.8%; *p* < 0.05).

## Discussion

We report the neurological symptoms of newly arrived refugees presenting to the ER of a large university hospital. This group of patients differs in several respects from the average patient population seen by European neurologists. Refugee patients presented with a spectrum of neurological disorders that is only partly explainable with cultural differences relative to the native patient population. We found a higher number of psychiatric diagnoses, in particular non-epileptic seizures, and a lower number of neurological emergencies requiring immediate in-hospital treatment. Moreover, most diagnoses mainly depended on thorough history taking rather than on physical examination.

Although recruited during the peak of refugee arrivals in Germany, the patient sample studied here is small, in particular in comparison to previous large studies of neurological disorders in refugee camps in non-European countries (11, 23, 24). The epidemiology of the populations in these studies differs importantly from our sample that consisted mostly of young male adults and did not include children and adolescents. Rather, our sample closely matches the demographical structure of immigrants as registered in 2015 in Greece and Italy, i.e., the main first destinations for refugees in Europe (25, 26). However, the unique social and political constellation in Germany in autumn 2015 allowed us to study whether forced migration affects the spectrum of neurological emergencies as seen in a European emergency department. These effects are usually difficult to disentangle from cultural influences on neurological symptoms (27, 28). We have therefore deliberately restricted our analysis to patients for whom medical records unequivocally documented an actual refugee status.

Consistent with previous studies, the most frequent neurological complaint on admission in all three groups was headache, with particularly high numbers in patients with immigrant background with and without refugee status (22, 29). Diagnoses differed however between groups. Refugees were mostly discharged with a diagnosis of headache of unknown etiology, whereas patient with migrational background and natives were mostly discharged with a diagnosis of migraine. This is obviously not the result of less thorough history taking or medical prejudices of emergency neurologists (30), as histories were longer in refugees and immigrant background patients compared to controls. Since both immigrant groups shared a similar cultural background and the prevalence of migraine in Arab countries does not differ from worldwide estimates (31), these differences are thus likely to result from difficulties in communication between emergency neurologist and patient.

The second most frequent complaint on admission in refugees were seizures or seizure-like symptoms. These symptoms occurred at a significantly higher frequency than in the other two groups. On the first glance, our results thus seem to confirm previous reports of a particularly high number of epilepsy patients in refugee camps outside Europe (10, 11). It has been speculated that this may reflect both an increased frequency of CNS disorders such as perinatal injury, genetic diseases, malnutrition, infectious diseases, previous stroke and direct consequences of war and conflict such as head trauma (23, 24, 32). The patient sample in our study did not present with an increased frequency of these disorders. Instead, we found a significant percentage of patients fulfilling the diagnostic criteria for “possible” or “probable” psychogenic non-epileptic seizures (33). The discrepancy to the aforementioned studies may point to the fact that refugees arriving in European countries often represent a selection of comparatively healthy young males that have received support from their families for migration to Europe (26). Conversely, children, women, older family members, and people with disorders of the CNS are less apt to cope with the stress and dangers of an—often illegal—journey to Europe. It is therefore not surprising that we found a low number of stroke patients in our sample, although cerebrovascular disease is considered to be a major neurological issue in refugee camps outside Europe (24).

Current pathogenetic concepts suggest that not only childhood trauma, but also trauma in adult life may be a precipitating factor that may trigger psychogenic non-epileptic seizures (34). Since the ethnic and cultural background of refugees and immigrant controls was similar, we deem a trauma-related mechanism relating to the causes for migration or to migration itself the likeliest explanation for the high number of psychogenic non-epileptic seizures. Together with other psychiatric diagnoses, this disorder accounted for 20% of all diagnoses on discharge. Conversely, none of the patients had presented with an established diagnosis or a suspicion of a psychiatric disorder on admission, thus suggesting that these diagnoses were established for the first time during the visit in the emergency department. It has been shown repeatedly that refugees frequently suffer from post-traumatic stress disorder (PTSD) and affective disorders (8, 9, 35). Moreover, there appear to be close pathophysiological links between psychogenic non-epileptic seizures and PTSD (34). For both disorders, early cognitive behavioral therapy is the mainstay of treatment (34, 36–38). Unfortunately, most neurological residents were not able to pave the way to appropriate and cultural competent follow-up care for refugee patients during the short stay in the emergency department.

Surveys in Western countries have repeatedly shown a great willingness of residents to care for diverse sociocultural patient groups, including immigrants and refugees [eg., (15, 39)]. However, most physicians also face major obstacles when caring for refugees, including language barriers, cultural differences, health concepts that differ from Western medicine, time constraints and lack of knowledge about medical disorders in the country of origin (9, 15, 39). Thus, many physicians do not feel sufficiently prepared for cross-cultural medical care (15, 39). Conversely, refugees often struggle with a lack of knowledge of the health system in Western countries, language barriers, lack of understanding of diagnostic tests and treatment recommendations and with difficult physician-patient relationships (9, 30). For example, in addition to a perceived lack of respect and valuation by some physicians, one repeated concern is that overly empathic physicians offer unjustified psychological explanations for physical complaints (30). Since we have no systematic follow-up data for our patients, it is indeed difficult to rule out definitely that the high percentage of psychiatric disorders is a result of misdiagnosis of primary neurological disorders, in particular epilepsy. On the other hand, our data are not indicative of any positive or negative bias toward refugee patients. Refugees were triaged like the other two patient groups, received the same attention during history taking and physical examination and underwent a similar diagnostic work-up, including cranial imaging. Full compliance with diagnostic procedures and therapeutic recommendations was observed with the same frequency as in the other two groups. However, our data also show that the entire management of neurological patients with refugee status nevertheless requires significantly more time and resources as in patients with immigrant background but established German residency and as in native Germans. Despite the ubiquity of English language in popular culture throughout the world, clinical management critically dependent on the presence of an interpreter in most cases, suggesting that low English proficiency contributed significantly to the total duration of stay in our emergency department. Despite the use of interpreters, significant uncertainties in communication remained that may have contributed to less MRIs/more CTs in refugees and a lower rate of immediate pharmacological treatment at discharge.

## Clinical Implications

The data presented here are a first neurological snapshot of a growing medical topic that will increasingly occupy neurologists in Western countries and that has hitherto not been part of resident training. Although the period investigated in our study may have been unique so far, the persistence of armed conflicts in the Great Middle East and other regions of the world increases the likelihood of further waves of forced migration. Neurologists must be aware that refugee patients present with a spectrum of emergencies and management problems that partly transcend the self-evident competences of physicians working in a multi-cultural metropolitan environment. They must be prepared to take responsibility for patients with neuropsychiatric diagnoses such as psychogenic non-epileptic seizures or PTSD that are likely consequences of conflicts and migration. These diagnoses need early and culturally competent follow-up interventions—also because they may present obstacles for integration in the country of resettlement. Emergency departments therefore need to take into account the resources that are required to organize further treatment and to reduce diagnostic uncertainties in refugees with neuropsychiatric disorders. This may be facilitated with the use of native-language questionnaires for the most frequent neurological complaints (e.g., headache, seizures, vertigo), trauma rating scales and the availability of professional interpreters. Standard operating procedures and contacts for subsequent qualified in- and out-of-hospital treatment should be available to emergency physicians and neurologists delivering primary care for this particular patient group. Most importantly, the perspective of refugees on medical care should be evaluated systematically and integrated into training of emergency neurologists.

## Data Availability Statement

The data supporting the conclusions of this manuscript will be made available by the authors to any qualified researcher.

## Author Contributions

MB was involved in study design, acquired all data, and drafted the manuscript. BvN performed statistical analyses. CL provided important methodological advice and revised the manuscript for intellectual content. CP designed and supervised the study and drafted the manuscript.

### Conflict of Interest Statement

The authors declare that the research was conducted in the absence of any commercial or financial relationships that could be construed as a potential conflict of interest.
